# Selective Laser Melting of Patient Individualized Osteosynthesis Plates—Digital to Physical Process Chain

**DOI:** 10.3390/ma13245786

**Published:** 2020-12-18

**Authors:** André Edelmann, Monique Dubis, Ralf Hellmann

**Affiliations:** Applied Laser and Photonics Group, University of Applied Sciences Aschaffenburg, 63743 Aschaffenburg, Germany; mo.dubis@gmail.com (M.D.); ralf.hellmann@th-ab.de (R.H.)

**Keywords:** digital process chain, titanium, medical implant, selective laser melting

## Abstract

We report on the exemplified realization of a digital to physical process chain for a patient individualized osteosynthesis plate for the tarsal bone area. Anonymized patient-specific data of the right feet were captured by computer tomography, which were then digitally processed to generate a surface file format (standard tessellation language, STL) ready for additive manufacturing. Physical realization by selective laser melting in titanium using optimized parameter settings and post-processing by stress relief annealing results in a customized osteosynthesis plate with superior properties fulfilling medical demands. High fitting accuracy was demonstrated by applying the osteosynthesis plate to an equally good 3D printed bone model, which likewise was generated using the patient-specific computer tomography (CT) data employing selective laser sintering and polyamid 12. Proper fixation has been achieved without any further manipulation of the plate using standard screws, proving that based on CT data, individualized implants well adapted to the anatomical conditions can be accomplished without the need for additional steps, such as bending, cutting and shape trimming of precast bone plates during the surgical intervention. Beyond parameter optimization for selective laser melting, this exemplified digital to physical process chain highlights the potential of additive manufacturing for individualized osteosynthesis.

## 1. Introduction

For medical applications, the additive manufacturing (AM) technology of selective laser melting (SLM) offers the distinguished opportunity of extensive customization as per individual patient data. This applies, e.g., for the fabricating of exact-fit, patient-specific orthopedic models, surgery-specific instruments and devices, artificial limbs and prosthetic implants [1,2,3,4]. As a consequence, medicine is one of the most rapidly growing areas of additive manufacturing in science, engineering and production [5]. The application of AM offers ideal fit of implants, allowing for fast and efficient production of customized products, thereby saving time and costs [6]. In addition, AM may also transform innovation and supply chain logistics for the medical device industry.

For the acquisition of patient-specific, individualized data, three dimensional imaging methods, such as optical 3D-scanners (laser scanning or stripe line projection), magnetic resonance imaging (MRI) and computer tomography (CT), provide fast, high resolution 3D views of patient anatomies [7,8]. Most commonly, a digital 3D CAD model is developed from MRI or CT imaging data by image post-processing and segmentation, and then exported into the STL format; finally, it is transferred to any additive manufacturing machine. The layer thickness may be decreased in slicing so as to increase the accuracy of the printing job and the finesse of the resulting implants [9].

The implications of AM for modern medical applications have been methodically reviewed in several journals—e.g., AM of calcium phosphate ceramics for orthopedic applications, such as bone grafting and drug delivery [10,11], with a particular focus on surgery, preoperative models for fractures and patient-specific surgical guides, and custom specific orthopedic implants [12,13]; regarding the applications in orthopedic surgery, see [14], and for the fabrication of orthopedic implants, see [15,16]. For a recent review on orthopedic implants, including both permanent joint replacements and temporary fracture fixation devices, such as the osteosynthesis plate addressed in this contribution, we refer to reference [17]. With respect to the response of human tissue upon implantation, several in vitro, ex vivo and in vivo studies have addressed questions of general materials’ biocompatibility, cell adhesion, proliferation and osteogenesis [16,18,19,20,21,22].

In view of selective laser melting (SLM), titanium and cobalt–chromium-based alloys particularly have been investigated for implants, dental and orthodontic applications, each of these materials having excellent mechanical properties and being considered as biocompatible for implantation [23,24,25,26,27]. In view of a coherent process chain from image acquisition to digital data processing and then to physical realization, interoperability of the digital process chain, which is defined as the ability of a numerical system to exchange data seamlessly, is highly desirable. Nowadays, however, the process chain is characterized by the use of different digital hardware systems and software solutions. As a result, current machine tool programming does not permit the transmission of high-level information through the entire digital process chain, leading to discontinuities in it. Though strong attempts have been undertaken to increase the degree of interoperability [28,29], most AM publications on medical applications focus on the optimization of process conditions and material and component properties. Though fully integrated process chains may not be conceivable today, the actual adaption of client data from CT to the manufacturing system with appropriate post-processing is rarely exemplified in the literature for concrete examples [30,31,32,33].

Against this background, in this contribution we demonstrate the generation of a patient-specific, individualized osteosynthesis plate by selective laser melting as to exemplify, beyond typically published parameter optimization for SLM, the clinical translation along the digital to physical process chain; i.e., the focus of this contribution is the exmeplification of the process chain, rather than optimization of materials or in vivo studies. Data organization of the medical images is done with the prevalent DICOM data format. The generation of a 3D model is performed by InVesalius, generating a STL in ASCII representation, which then can be further edited by Autodesk MeshMixer for repair and reduction and by Autodesk Mesh Enabler to generate volume or surface models. Conversion of the individual CT sectional images into the STL format is done with a sagittal cut, allowing high resolution. Physical realization was performed by selective laser mating using titanium grade 4 for medical implant applications, as has been qualified for good osteointegration.

## 2. Material and Methods

### 2.1. Selective Laser Melting Conditions

**Figure 1:**
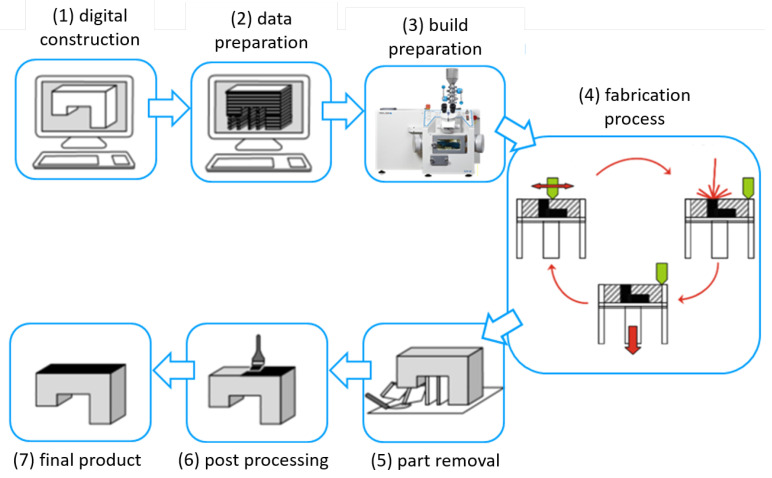
Fabrication process in selective laser melting.

For selective laser melting we employed a Realizer SLM 50 (Realizer GmbH, Borchen, Germany) equipped with a $P_{L}=100$ W yttrium fiber laser (IPG, YLM-100). The spot size of the laser was set to $d_{\mathrm{spot}}=20$ μm at focus position. The machine processes under an argon atmosphere with a 0.1% oxygen level. The platform was kept at $\vartheta_{\mathrm{plate}}=200$ °C to maintain the machined part at an elevated temperature to avoid deformation by curling due to residual stress. In Figure 1 the physical process chain in selective laser melting is shown. The physical process chain consists of the following steps: (1) digital construction of the part, (2) data preparation for processing in additive manufacturing—e.g., defining the build orientation and support structures, (3) preparation of the AM system, (4) AM fabrication process, (5) part removal from the AM system—e.g., part cutting from the build plate and thermal treatment, (6) post-processing of the surface—e.g., blasting and (7) final product ready for application [34,35,36].

**Figure 2:**
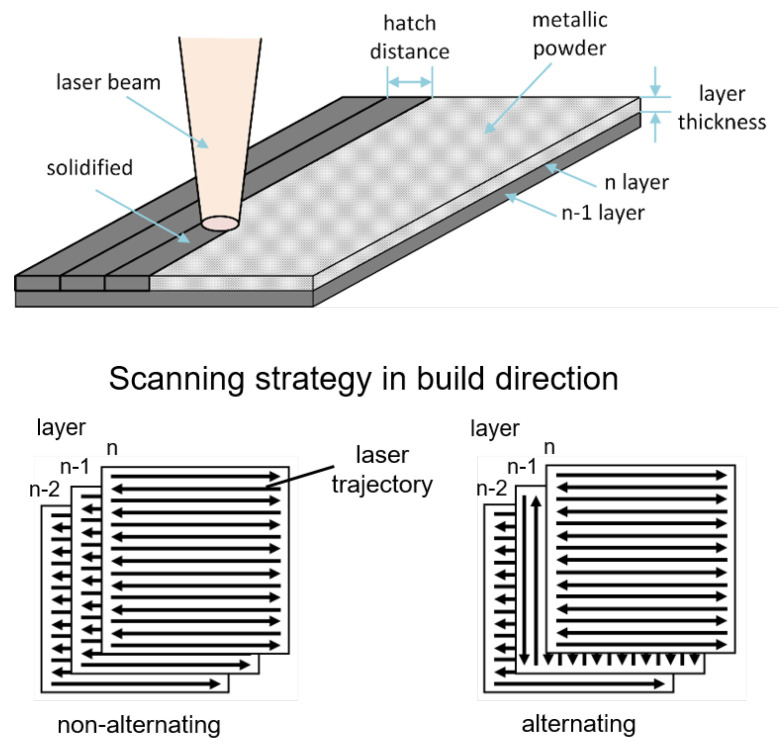
Laser processing of metallic powder (**top**) and scanning strategy in build direction (**below**).

The laser processing of the metallic powder is schematically shown in Figure 2 (top). To solidify the powder, the laser scans on linear trajectories spaced by the hatch distance across the powder bed. The process is finished by scanning a contour line around the processed area. The scanning strategy for the hatch can be varied in build direction. In Figure 2 (below), non-alternating and alternating scanning strategies are illustrated. Using a non-alternating scanning strategy, the hatch trajectory in each layer remains constant. In contrast, applying an alternating scanning strategy, the hatch trajectory is rotated by $90^{{}^{\circ}}$ in each layer. The surface quality, i.e., the surface roughness and scalloping of the surface, can be influenced by the applied scanning strategy.

### 2.2. Powder Characteristics and Mechanical Properties

Important material parameters for processing metallic powder in SLM systems are the flowability and the powder density [37,38,39,40]. If the flowability is too low, a homogenous coverage of the powder across the build plate in the SLM machine cannot be ensured, and in addition, a low powder density can lead to a higher porosity of the fabricated components [41]. The flowability, which influences the coating of the powder bed, was determined by measuring the flow rate using a Hall-Flowmeter according DIN EN ISO 4490. For the titanium powder used in our study, a flowability of 31s was measured, allowing for a homogeneous powder bed in the SLM process.

The powder density, influencing the homogeneity and density of the powder bed and thus the accessible relative density of the SLM built part, was measured according DIN ISO 697 to be about 2.5 g/cm^3^. The moisture of the powder, which reduces the flow rate and thus the coating of the powder bed and which may also lead to higher gas porosity, was determined by weighting in combination with a drying chamber (PCE-MA 110, PCE Germany GmbH, Meschede, Germany). We determined the moisture of the titanium powder to be below 0.01%, and therefore, excluded any influence of moisture on the flowability and powder density.

Besides depending on the powder characteristics, the part quality (e.g., porosity and surface roughness) depends also on the applied process parameters. We thus have optimized the process parameters for the area, contour line and support structure by varying the laser power $P_{L}$ and hatch distance, while keeping the layer thickness $t_{\mathrm{layer}}=25$ μm and scan velocity $v_{\mathrm{scan}}=250$ μm constant. The optimized process parameters are listed in Table 1.

The resulting mechanical properties of the additive manufactured specimens were evaluated with respect to the build orientation and were compared to the requirements of medical implants according the standard DIN 5832-2. All specimens were heat treated using a heating rate of 10 K/min up to 550 °C with 60 min of holding time, followed by natural cooling (LH 120, Nabertherm, Lilienthal, Germany). The arithmetic means of the results of the mechanical tests are listed in Table 2. The specimens for tensile strength tests were fabricated in the build orientations of $0^{{}^{\circ}}$, $45^{{}^{\circ}}$ and $90^{{}^{\circ}}$ with respect to the build plate. We evaluated four specimens for each build direction (AG-Xplus, Shimadzu, Ky$\bar{o}$to, Japan). For all orientations, the measured tensile strength and failure strain were above the requirements of DIN 5832-2. The highest value of the tensile strength was found for the build orientation of $0^{{}^{\circ}}$. The values for $45^{{}^{\circ}}$ and $90^{{}^{\circ}}$ are close to each other. For the failure strain, only small variations were found for the different build orientations. The Vickers hardness was measured on the top surfaces of heat treated cubic specimens (edge size 1 cm). We used four specimens and carried out 15 measurements for each specimen (Falcon 5000, Innovatest, Maastricht, The Netherlands). The arithmetic mean of the Vickers hardness resulted in HV5=240±7.8 kgf. The remaining porosity of the specimens was measured by Archimedes’ method of below σ=0.3% (arithmetic mean over 5 measurements).

### 2.3. Wetting Characteristics

**Figure 3:**
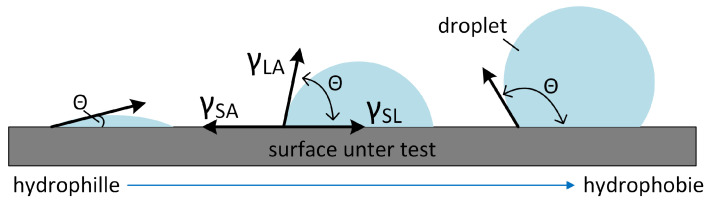
Surface wetting characteristic and relation to the contact angle.

The wetting behavior of technical surfaces can be characterized by the contact angle of a droplet [42]. The method of Young [43] used for smooth surfaces is shown on Figure 3.

By the equilibrium relation between the interfacial tensions of $\gamma_{\mathrm{LA}}$ (solid–gaseous), $\gamma_{\mathrm{SA}}$ (liquid–gaseous) and $\gamma_{\mathrm{SL}}$ (solid–liquid), the contact angle Θ of the droplet can be calculated by [42].

$$\cos\left(\Theta \right)=\frac{\gamma_{\mathrm{SA}}-\gamma_{\mathrm{SL}}}{\gamma_{\mathrm{LA}}}.$$

A surface with contact angle of smaller than $90^{{}^{\circ}}$ is called hydrophilic, and above $90^{{}^{\circ}}$, hydrophobic. For a removable osteosynthesis plate a hydrophobic characteristic is desired to avoid an engraftment of the implant. The measurements were carried out by the optical contact angle measurment system OCA 20, DataPhysics Instruments, Filderstadt, Germany.

### 2.4. Optical Characterization Tools

The shape deviation and surface roughness were measured by optical characterization tools, the former being obtained by 3D inspection (3D scanning) of the implant using stripe line projection employing Atos Core 3D- scanner (GOM, Braunschweig, Germany). Geometrical deviations between the digitally designed (CAD data) and physically realized implant, i.e., verification of the shape accuracy, were revealed by shape variance analysis using ATOS Professional V8-SR1 software (GOM, Braunschweig, Germany). The surface roughness was analyzed by the optical 3D-profilometer VR-3200 (Keyence, Osaka, Japan). The surface profile was measured by stripe line projection, and the arithmetic mean value of the surface roughness Sa was calculated.

## 3. Results and Discussion

### 3.1. Digital Process Chain

**Figure 4:**
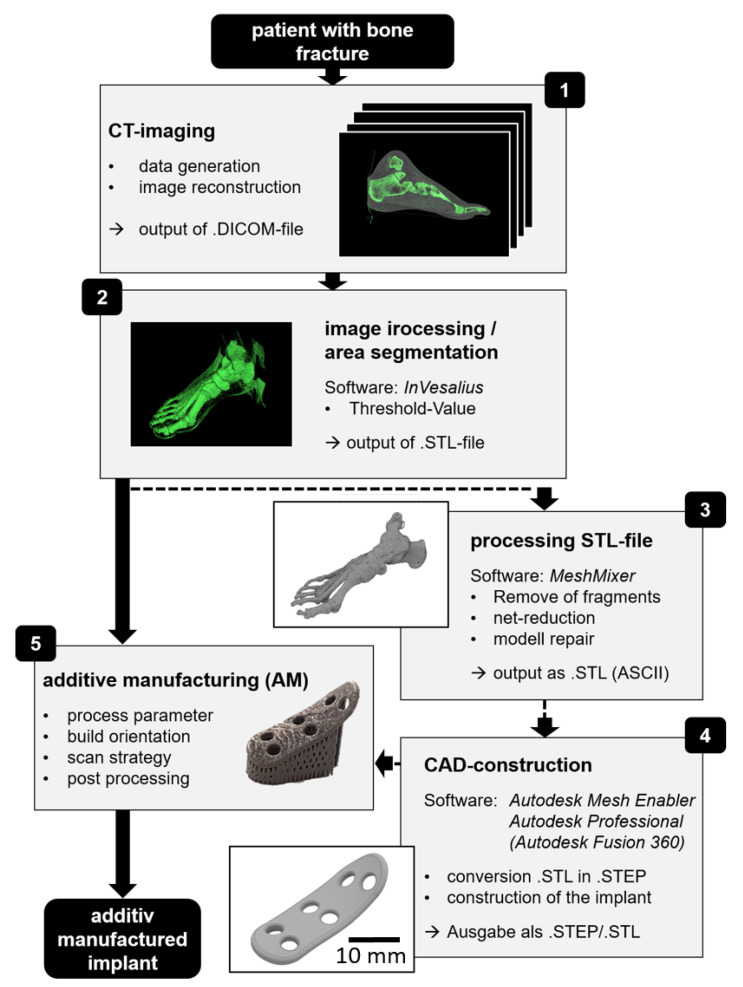
Digital process chain.

In additive manufacturing systems, the standard input data are obtained from an .stl file, describing the surface of the part by small triangles. The .stl-file is generated by the CAM system after digital construction of the part or by 3D scanning of the surface of an existing part. For designing an individual implant, an adaption of the geometry of the implant to the bone is needed. The digital process chain of the fabrication of an individual implant is shown in Figure 4. The original data were obtained by CT-imaging of the human metatarsal area. The 3D image was obtained by a composition of sequential 2D images, available in the .DICOM data format (see point 1 in Figure 4). To obtain the surface data from the metatarsal bone, we have to combine the sectional images from the CT to a 3D image and to separate the human tissue from the bone. The image processing and area segmentation were done by the software InVesalius and resulted in a surface model of the metatarsal bone represented by an .stl file (see point 2 in Figure 4). We used the Hounsfield scale (HU) to separate the bone form tissue with a specific threshold [44]. Standard-values of the Hounsfield scale, are e.g., 0 HU for water and 350 to 2000 HU for bones (cortical). The software system In Veaslius was used for separation and identification of the bone areas and to convert the CT sectional imaging into a .stl data format.

The output .stl-file from the InVesaluis software contains open surface areas and consists of a high amount of data points. The software Autodesk Meshmixer was used to close all open surface areas and to reduce the data points without significant quality loss of the structure (see point 3 in Figure 4). To construct the individual implant, we then used the software Autodesk Mesh Enabler to generate a volume model in the .step data format. In this software system, an adapted free form plate matching the bone structure can be generated. In Autodesk Mesh Enabler, the bone’s surface is divided into small areas; the sections in between are then interpolated by splines, forming a free-form surface contour plate. The free-form plate is imported into an Autodesk Inventor to generate the final contour and place the screw holes into the plate. Finally, we obtain the individualized digital implant in .step data format (see 4. point in Figure 4). For the fabrication of the physical implant, we used the additive manufacturing technology of selective laser melting, as described in the following sections.

### 3.2. Fabrication Accuracy and Thermal Treatment

**Figure 5:**
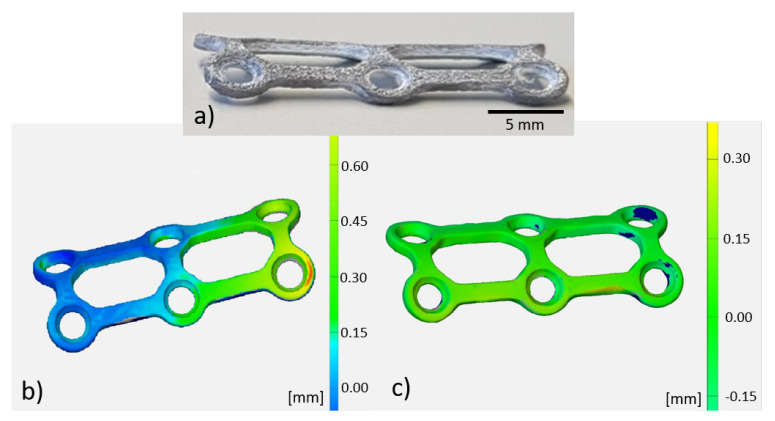
(**a**) AM fabricated implant and nominal/actual value comparison of (**b**) as-built and (**c**) after thermal treatment.

A fabricated standard implant as built, i.e., without any further post-processing, for bone fractures using SLM technology, is shown in Figure 5a. We measured shape deviations and shape variance analysis as compared to the original CAD data, as provided by striped line projection using Atos Core and ATOS Professional V8-SR1 software. In Figure 5b the result is shown for the as-built implant and in Figure 5c after the thermal treatment. Significant smaller deviations were found after the thermal treatment.

### 3.3. Surface Structuring and Wetting Characteristics

**Figure 6:**
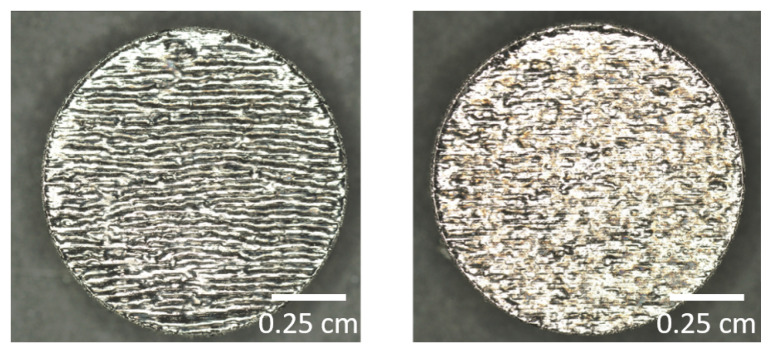
Top surface of additive manufactured specimen using not-alternating (**left**) and alternating (**right**) scanning strategies.

As osteosythesis plates are supposed to be removed after a certain healing period, the surface properties resulting from the SLM process that are assertive for the engraftment of tissue were studied. Therefore, different surface textures were produced by SLM processing. Initially, we analyzed a layer-wise alternating scanning strategy of $90^{{}^{\circ}}$ and a non-alternating ($0^{{}^{\circ}}$) scanning strategy. Using a not-alternating (na) scan-strategy, periodic groves on the top surface appear, whereas an alternating scanning strategy of $90^{{}^{\circ}}$ leads to a stochastic surface profile. In Figure 6, microscopic images of both surface structures are shown.

**Figure 7:**
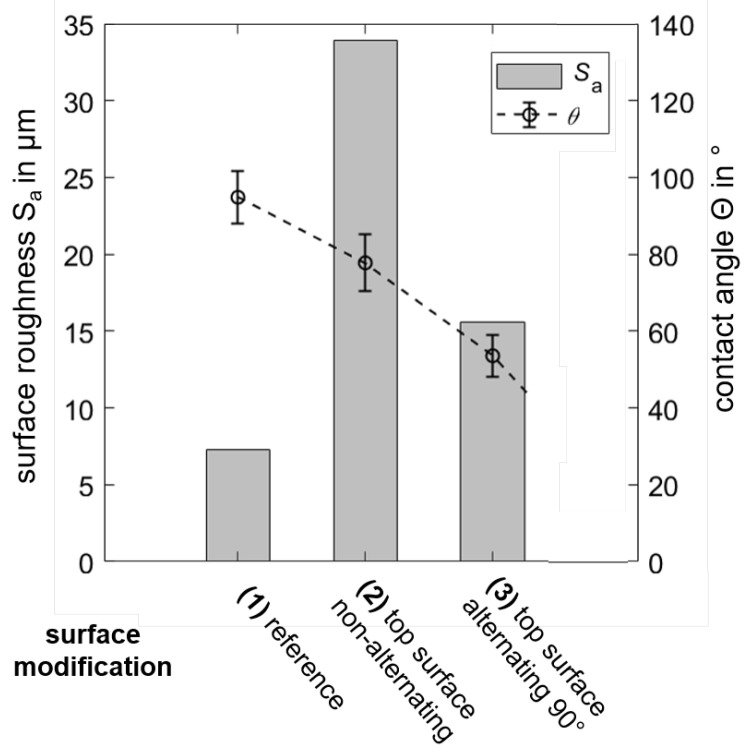
Surface roughness and contact angle (wetting characteristics) of different surface conditions of the additively manufactured implant.

To evaluate these surfaces, their roughness and wetting characteristics were measured by an optical 3D-profilometer and a contact angle measurement system, and compared to a commercially available, polished implant, denoted as reference (1). Figure 7 summarizes the results for different surface conditions of the reference and SLM fabricated specimens using non-alternating (2) and alternating (3) scanning strategies. Here, the used specimens are small disks with a diameter of d=10 mm and a thickness of t=2 mm, respectively.

By comparing the surface roughness $S_{a}$ and contact angles Θ for alternating and non-alternating scanning strategies, both $S_{a}$ and Θ apparently decrease when applying the alternating scanning strategy. Specifically, the surface roughness drops by $\Delta S_{a}=43\;\mu m$ to about $S_{a}=15\;\mu m$ and the wetting angle by $\Delta \Theta =60^{{}^{\circ}}$. These values were measured directly after SLM fabrication without any post-processing of the surface. Sand blasting can reduce the surface roughness by about 15%, reaching values of about 11μm [45] for titanium, yet being higher than the surface roughness of the reference, which was polished.

For non-alternating and alternating scanning strategies, the contact angle Θ, measured on the day of production of the SLM-parts, is significantly smaller than Θ of the reference and decreases due to the different surface texture for alternating scanning. However, as it has previously been reported, laser structured metal surfaces alter the wetting behavior with a temporal evolution of the apparent contact angle over days [46,47]. We found that after 14 days of storage at normal ambient conditions, Θ increased by about 25–30%, depending on the applied hatch distance; i.e., wettability moves towards higher hydrophility, closing the gap toward the reference wettability. These results indicate that the alternating scanning strategy is to be preferred for fabrication of the implant. Here the lowest value of surface roughness was found, and for the contact angle we expected a further increase after 14 days of storage.

### 3.4. Implant Fabrication

**Figure 8:**
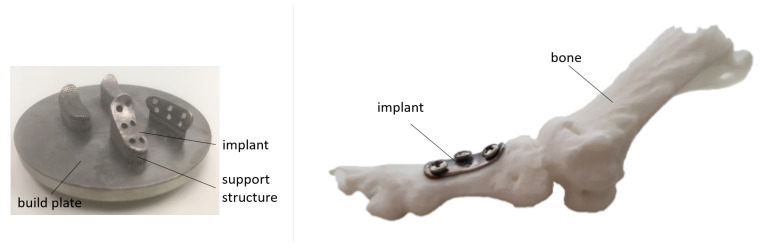
(**Left**): Build plate (diameter $d_{\mathrm{plate}}=80$ mm) with implants in different orientations. (**Right**): Sector of the 3D printed metatarsal bone area with additively manufactured implant.

For exemplification of the digital and physical process chain, an individual metatarsal bone was fabricated. The digital data were gained from CT-imaging of an anonymized patient. The metatarsal bone implant was designed as shown in Section 3.1 and was thus adapted to the desired bone surface. After designing the final implant, an .stl output file was generated, which contains the surface model of the individual implant and was used as input data format of the SLM system. The metatarsal bone implant was fabricated using Ti4-powder and post-processed using stress relief annealing, as discussed above. The fabricated implant on the build-plate, directly after the removal from the AM system, is shown in Figure 8 (left).

For a fitting test of the individual implant, we have also reconstructed and 3D printed the metatarsal bone using selective laser sintering technology and polyamid 12 (PA 12). For the reconstruction of the bone, the data from CT-imaging (cf. step 2 of the digital process chain in Figure 4) were used to generate a plastic duplicate of the bone. As shown in Figure 8 (right), the implant was fixed on the desired bone area using standard screws; proper fixation was achieved without any further manipulation of the plate, such as bending or shape trimming of precast bone plates, which is, in turn, today typically employed in standard surgical intervention. This proves and highlights that based on CT data, individualized implants made by SLM can be well adapted to the individualized anatomical conditions.

## 4. Conclusions

We described and exemplified a digital to physical process chain for additive manufacturing a patient-specific component for plate osteosynthesis in the tarsal bone area. Starting from anonymized patient-specific data captured by computer tomography, a sequence of data processing tools using commercially available software tools and finally feeding a selective laser melting machine tool led to the physical realization of titanium-based, customized plate osteosynthesis with high fitting accuracy. The specimen fulfilled the mechanical requirements for medical components, as determined by the Vickers hardness of HV5 = 240 kgf, tensile strength of $\sigma_{m}$ = 561–654 $N/{\mathrm{mm}}^{2}$ and failure strain between ε = 17–19%.

Beyond often published parameter optimization of selective laser melting, this exemplified digital to physical process chain highlights the potential of additive manufacturing for individualized osteosynthesis.

## Figures and Tables

**Table 1:** Process parameters.

	Energy Density (J/${\mathrm{mm}}^{3}$)	Laser Power (W)	Hatch Distance (μm)
area	171	85.6	0.08
contour	96	30.2	0.05
support	6	35.8	—

**Table 2:** Mechanical properties of additive manufactured part (as-built) compared to DIN 5832-2.

	DIN 5832-2	$0^{{}^{\circ}}$	$45^{{}^{\circ}}$	$90^{{}^{\circ}}$
tensile strength [N/mm^2^]	550	654±27	561±5	568±11
failure strain [%]	15	19.1±1.0	16.3±1.1	18.9±0.3
